# Bioinformatic analysis of peripheral blood miRNA of breast cancer patients in relation with anthracycline cardiotoxicity

**DOI:** 10.1186/s12872-020-01346-y

**Published:** 2020-02-03

**Authors:** Wang Yadi, Chen Shurui, Zhang Tong, Chen Suxian, Tong Qing, He Dongning

**Affiliations:** 1 The Third Affiliated Hospital, Jinzhou Medical University, Jinzhou, China; 2 Jinzhou Medical University, Jinzhou, China; 3 TongHua Dongbao pharmaceutical Co., Ltd, Tonghua, China; 4 Department of Oncology, The First Affiliated Hospital of Jinzhou Medical University, Jinzhou, China

**Keywords:** Bioinformatics, Breast cancer, Cardiotoxicity, miRNA, Signaling pathway

## Abstract

**Background:**

The current diagnostic methods and treatments still fail to lower the incidence of anthracycline-induced cardiotoxicity effectively. In this study, we aimed to (1) analyze the cardiotoxicity-related genes after breast cancer chemotherapy in gene expression database and (2) carry out bioinformatic analysis to identify cardiotoxicity-related abnormal expressions, the biomarkers of such abnormal expressions, and the key regulatory pathways after breast cancer chemotherapy.

**Methods:**

Cardiotoxicity-related gene expression data (GSE40447) after breast cancer chemotherapy was acquired from the Gene Expression Omnibus (GEO) database. The biomarker expression data of women with chemotherapy-induced cardiotoxicity (group A), chemotherapy history but no cardiotoxicity (group B), and confirmatory diagnosis of breast cancer but normal ejection fraction before chemotherapy (group C) were analyzed to obtain the mRNA with differential expressions and predict the micro RNAs (miRNAs) regulating the differential expressions. The miRanda formula and functional enrichment analysis were used to screen abnormal miRNAs. Then, the Gene Ontology (GO) analysis was adapted to further screen the miRNAs related to cardiotoxicity after breast cancer chemotherapy.

**Result:**

The data of differential analysis of biomarker expression of groups A, B, and C using the GSE40447-related gene expression profile database showed that there were 30 intersection genes. The differentially expressed mRNAs were predicted using the miRanda and Target Scan software, and a total of 2978 miRNAs were obtained by taking the intersections. Further, the GO analysis and targeted regulatory relationship between miRNA and target genes were used to establish miRNA-gene interaction network to screen and obtain seven cardiotoxicity-related miRNAs with relatively high centrality, including hsa-miR-4638-3p, hsa-miR-5096, hsa-miR-4763-5p, hsa-miR-1273 g-3p, hsa-miR6192, hsa-miR-4726-5p and hsa-miR-1273a. Among them, hsa-miR-4638-3p and hsa-miR-1273 g-3p had the highest centrality. The PCR verification results were consistent with those of the chip data. There are differentially expressed miRNAs in the peripheral blood of breast cancer patients with anthracycline cardiotoxicity. Among them, hsa-miR-4638-3p and hsa-miR-1273 g-3p are closely associated with the onset of anthracycline cardiotoxicity in patients with breast cancer. The signaling pathway is mainly concentrated in TGF-β signaling pathway and adhesion signaling pathway.

**Conclusions:**

Changes in expression of hsa-miR-4638-3p and hsa-miR-1273 g-3p may contribute to the detection of anthracyclines induced cardiac toxicity, and their potential function may be related to TGF-β signaling pathway and adhesion signaling pathway.

## Background

Combined treatment based on anthracyclines, such as doxorubicin, epirubicin, and pirarubicin, is usually the standard regimen for the first-line treatment of breast cancer. It has definite therapeutic effect and is indispensable. However, cardiotoxicity is the most serious side effect of anthracyclines. Both clinical research and practical observations have shown that cardiotoxicity induced by anthracyclines is often progressive and irreversible [1, 2]. Cardiac injury might occur even when anthracyclines are used for the first time. Therefore, it is particularly important to actively prevent the cardiotoxicity induced by anthracyclines. Currently, the therapeutic effect on patients with anthracycline-induced cardiotoxicity is highly limited. The current diagnostic methods and treatment levels still fail to lower the incidence of anthracycline-induced cardiotoxicity effectively. This is because anthracycline-induced cardiotoxicity is a complicated, multifactorial, and multistep biological process. The existing research achievements are not sufficient to fully reveal the mechanism of its incidence and development. It is important to identify methods for the early specific diagnosis and effective prognosis.

During recent years, the use of high-throughput methods to detect gene expression has become a common practice. Microarray chip technology can quantify tens of thousands of gene transcript information simultaneously. The gene expression omnibus Gene Expression Omnibus (GEO) is currently the largest public high-throughput molecular abundance expression database globally. It mainly stores gene expression data. The GEO allows researchers to upload, download, save, and retrieve different types of genomic data.

In the present study, we aimed to (1) analyze the cardiotoxicity-related genes after breast cancer chemotherapy in gene expression database and (2) carry out bioinformatic analysis to identify cardiotoxicity-related abnormal expressions, the biomarkers of such abnormal expressions, and the key regulatory pathways after breast cancer chemotherapy.

## Methods

### Data mining and analysis

#### GEO database mining and chip dataset acquisition

The microarray expression profile dataset GSE40447 deposited by McBCaffrey et al [3], was downloaded from the Gene Expression Omnibus (GEO) database (https://www.ncbi.nlm.nih.gov/geo/query/acc.cgi?acc=GSE40447). The chip was from the Affymetrix human genome U133A platform, containing 22,283 gene probes. and included patients with confirmatory diagnosis of breast cancer: women with anthracycline-induced cardiotoxicity, chemotherapy history but no cardiotoxicity, and normal ejection fraction before chemotherapy. According to the test results, the raw data in these chips were divided into group A (female breast cancer patients with anthracycline-induced cardiotoxicity), group B (female breast cancer patients with chemotherapy history but no cardiotoxicity), and group C (female breast cancer patients with normal ejection fraction before chemotherapy, that is Group D in the original text [3]).

#### Data preprocessing and differential expression analysis

For each sample in GSE40447 data, the gene expression value of all the probes was reduced to a relatively absolute average gene expression value. Subsequently, we estimated the missing data and carried out quantile normalization of the data. The R-value of the differentially expressed genes was determined. To avoid false positive results caused by multiple test problems, Benjamin Hochberg method was used to correct the primary *P*-value to the False Discovery Rate (FDR). The FDR < 0.05 was adopted as the cut off.

#### GO analysis and DEGs analysis

For GSE40447 data, The Gene Ontology (GO) analysis is used to generate a dynamic controlled vocabulary (character table) that can be applied to all studies on eukaryotic genomes. It is often used to study and analyze large-scale genome and transcriptome data [4]. The Differentially Expressed Genes (DEGs) data were divided into two groups—the up-regulated and down-regulated groups based on gene overexpression [5]. Subsequently, the GO analysis was performed using the gene set analysis toolkit. The difference was considered statistically significant when *P <* 0.05.

#### Enrichment analysis

Regarding the functional annotation of Differentially Expressed Genes (DEGs) in GSE40447 data, the enrichment expression of Kyoto Encyclopedia of Genes and Genomes (KEGG) pathway has been defined by KAAS [6]. The difference was considered statistically significant when *P < 0.05*.

#### miRNAs expression detection by real-time quantitative PCR

Our laboratory research sample: 20 patients and 40 samples (20 before and 20 after chemotherapy) of frozen blood of patients with breast cancer that was collected before and after chemotherapy respectively and stored in low-temperature refrigerators (− 80 °C) were randomly sampled from the First Affiliated Hospital and Third Affiliated Hospital of Jinzhou Medical University from May 2017 to August 2018. The purpose of this study was to compare the results of our selected study samples with online available published data.

Reagents: The miRcuit hsa-miRNA isolation kit (Cat# DP501), miRcuit hsa-miRNA First-Strand cDNA Synthesis Kit (Cat# KR201) and miRcuit hsa-miRNA Detection Kit (SYBR Green) (Cat# FP401) was purchased from TransGen Biotech (Beijing,China); Water-DEPC treated from Beijing Solarbio Science & Technology Co., Ltd. (Beijing,China); and the miRNA primers were designed and synthesized by Changchun Jing Mei Biological Engineering Co. Ltd. (Changchun, China).

Instruments: The Nanodrop 2000 ultra-micro spectrophotometer (Thermo, USA), Stepone quantitative PCR instrument (ABI, USA); and Bioptic Qsep100 automatic nucleic acid analyzer (BiOptic, Taiwan).

#### RT and q-PCR detection of miRNA expression

The miRNA extracted from the plasma were reverse transcribed into cDNA according to the instructions provided in the Takara reverse transcription kit. The corresponding RT primers were then added. The subsequent quantitative-polymerase chain reaction (q-PCR) was carried out using the SYBR Green PCR kit with the synthesized cDNA as the template. The reaction conditions were as follows: denaturing at 94 °C for 2 min, followed by heating at 94 °C for 20 s and 60 °C for 34 s with a total of 40 cycles. Using U6 as the reference, the Ct value, dissolution curve, and amplification curve of the samples were analyzed after the experiment. The following primer sets were used for RT-PCR: miR4638-3P:TGGACACCGCTCAGCCGG; miR1273g-3P:ACCACTGCACTCCAGCCTGAG; U6:CTCGCTTCGGCAGCACA.

### Statistical analyses

The SPSS 17.0 statistical software was used for data analysis. The measurement data with normal distribution are expressed as mean *±* standard deviation (*X ±* SD*)*, and paired *t* test was used for comparison between groups. The measurement data with non-normal distribution are expressed as median (interquartile range, IQR), and Wilcoxon rank sum test was used for comparison between groups. The χ^2^ test was used to compare the enumeration data between the groups. The difference was statistically significant when *P <* 0.05.

## Results

### DEGs and analysis of their biological functions

A total of 19 cases (5 cases for group A, 10 cases for group B, and 4 cases for group C) were found to be qualified for the study after strict filtering and screening of the GSE40447 data. Among the three groups, the 19 blood samples were assessed. Compared with that of group B, group A had 2282 DEGs, including 1321 up-regulated DEGs and 961 down-regulated DEGs. Compared with that of group C, group A had 1195 DEGs, including 752 up-regulated DEGs and 443 down-regulated DEGs. Compared with that of group C, group B had 1718 DEGs, including 525 up-regulated DEGs and 1193 down-regulated DEGs. The DGE analysis among groups A, B, and C showed that there were 30 intersection genes (Table 1).

**Table 1:** List of intersection genes with differential gene expression in groups A, B, and C

Number	Gene symbol	A vs. B FC	A vs. C FC	B vs. C FC	
1	MAPK1	1.33	1.39	−1.46	
2	NPHP3	1.33	1.34	1.49	
3	ARL17A	−1.62	−1.67	1.41	
	ARL17B	−1.62	−1.67	1.41	
	LOC100294341	−1.62	−1.67	1.41	
	LOC100996709	−1.62	−1.67	1.41	
4	MBP	1.51	1.55	1.43	
5	RABGAP1L	−1.23	−1.28	−1.55	
6	PHLDB2	**−1.51**	**1.24**	**1.69**	
7	PECAM1	1.35	1.26	1.42	
8	PVT1	−1.35	−1.27	− 1.23	
9	ITGB8	**1.41**	**−1.37**	**−1.41**	
10	LIPJ	1.30	1.26	1.29	
11	ATXN2L	1.62	1.63	−1.44	
12	TCEB3	1.48	1.45	−1.42	
13	CTIF	1.57	1.42	1.22	
14	OSBPL1A	1.47	1.29	−1.37	
15	LINC01590	**−1.65**	**1.45**	**1.58**	
	SMIM8	**−1.65**	**1.45**	**1.58**	
16	PLCB1	1.22	1.29	1.56	
17	CENPT	**1.37**	**−1.37**	**−1.35**	
18	ENGASE	1.27	1.68	1.33	
19	FAM27B	1.36	2.22	1.64	
	FAM27C	1.36	2.22	1.64	
	LOC102725186	1.36	2.22	1.64	
	LOC105379444	1.36	2.22	1.64	
20	SPTBN1	−1.56	−1.33	1.54	
21	VPS53	**1.29**	**−1.58**	**−1.31**	
22	RNF34	1.32	1.54	−1.36	
23	PGM5-AS1	**−1.31**	**2.00**	**1.34**	
24	FGFR1OP2	−1.47	−2.28	−2.27	
25	GKAP1	**−1.67**	**1.42**	**1.29**	
26	ZNF736	**−1.79**	**1.27**	**1.36**	
27	TTC26	**−1.79**	**1.42**	**1.73**	
28	TSHZ2	−1.79	−2.20	− 1.66	
29	DUSP8	1.39	1.47	1.30	
30	GPATCH2	**−1.6**	**1.24**	**1.29**	

Note: group A: female breast cancer patients with anthracycline-induced cardiotoxicity; group B (female breast cancer patients with chemotherapy history but no cardiotoxicity; group C: female breast cancer patients with normal ejection fraction before chemotherapy. Bold font indicates that the trends of the gene in group A were opposite to those in groups B and C. Red represents up-regulation of group A genes and down-regulation of groups B and C genes; green represents down-regulation of group A genes and up-regulation of groups B and C genes

*FC* Fold Change, *PHLDB2* pleckstrin homology like domain family B member 2, *LINC01590* long intergenic non-protein coding RNA 1590, *SMIM8* small integral membrane protein 8, *CENPT* centromere protein T, *VPS53* VPS53 subunit of GARP complex, *ITGB8*:integrin subunit beta 8, *TTC26* tetratricopeptide repeat domain 26, *PGM5-AS1*:PGM5 antisense RNA 1, *GKAP1* G kinase anchoring protein 1; *ZNF736*:zinc finger protein 736; *GPATCH2*:G-patch domain containing 2;

Entries in boldface font indicates that the trends of the gene in group A were opposite to those in groups B and C

### GO and signaling pathway analyses

**Fig. 1:**
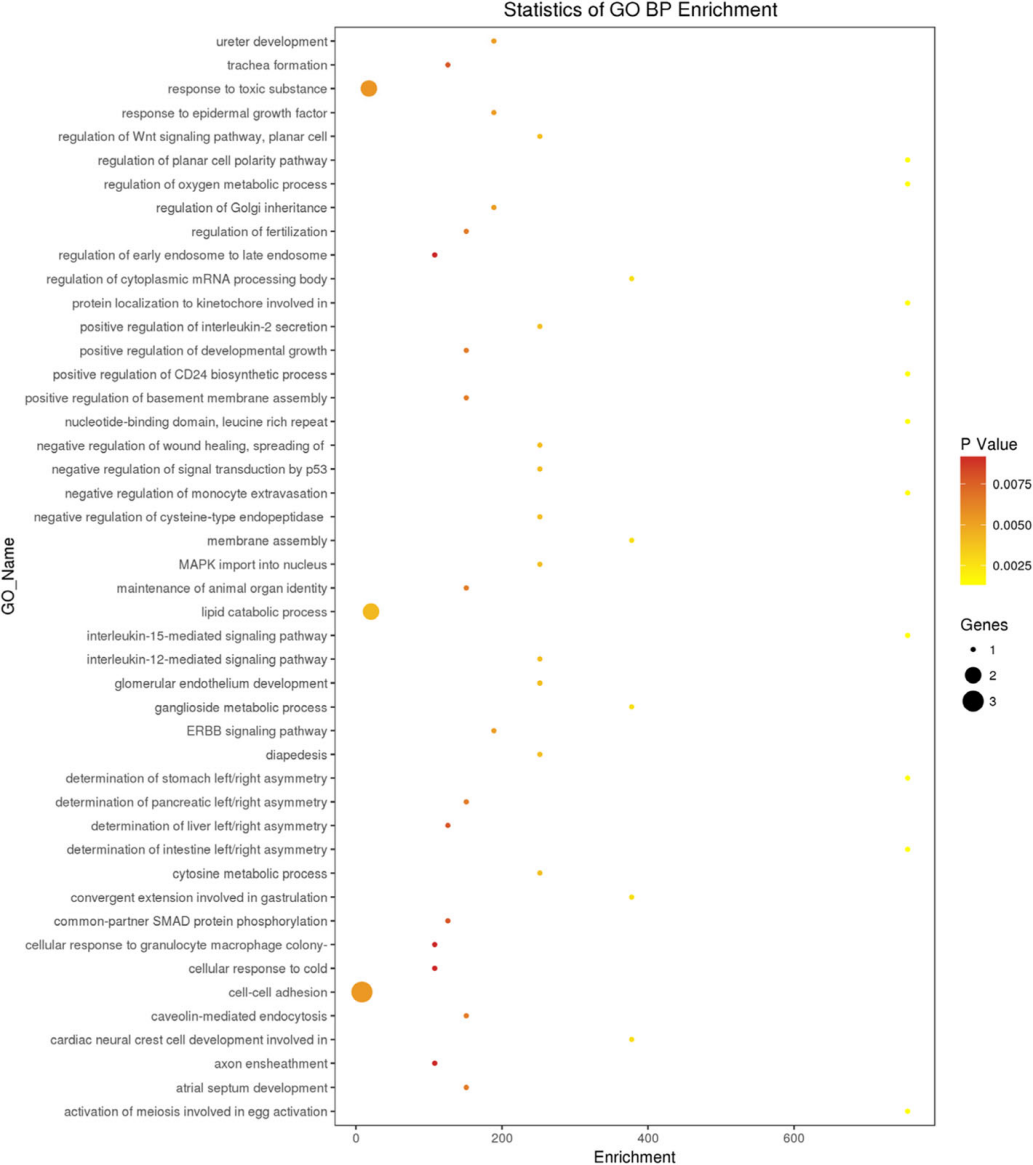
Biological functions of the differentially expressed intersection genes. Note: BP:Biological Process. The x-axis represents the number of DEGs and the y-axis represents the GO terms. The graph displays only significantly enriched GO terms (*P* < 0.05) and darker yellow indicates greater significance

**Fig. 2:**
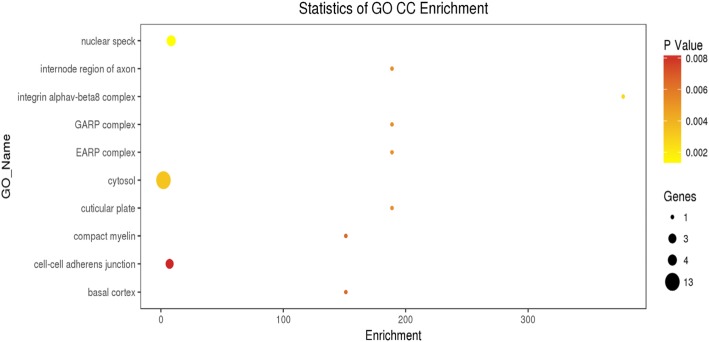
Cellular components of the differentially expressed intersection genes. Note: CC:Cellular Component. The x-axis represents the number of DEGs and the y-axis represents the GO terms. The graph displays only significantly enriched GO terms (*P* < 0.05) and darker yellow indicates greater significance

**Fig. 3:**
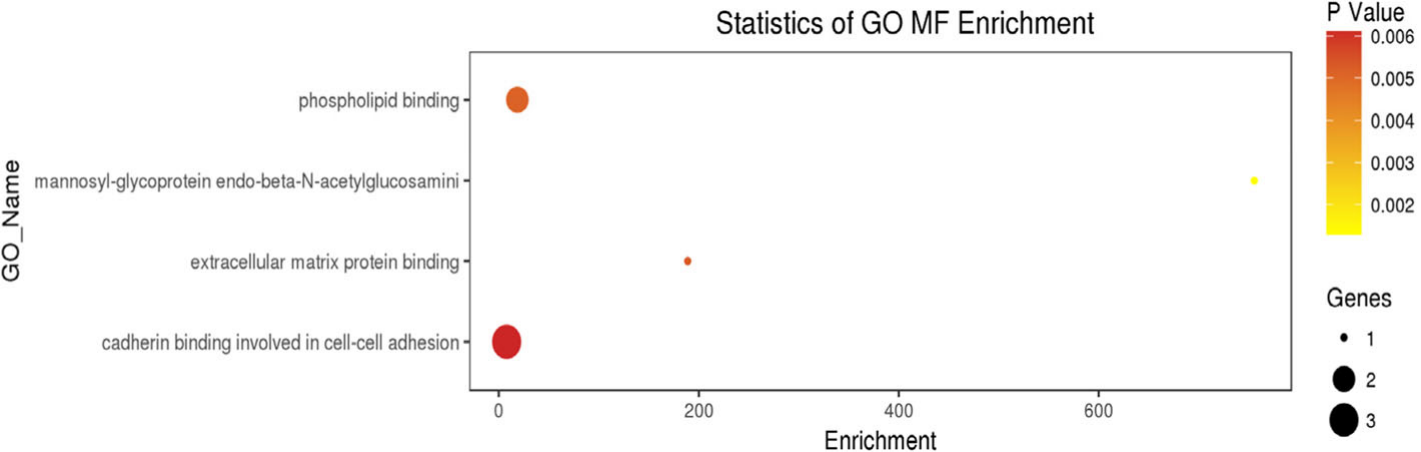
Molecular functions of the differentially expressed intersection genes. Note: MF:Molecular Function. The x-axis represents the number of DEGs and the y-axis represents the GO terms. The graph displays only significantly enriched GO terms (*P* < 0.05) and darker yellow indicates greater significance

**Fig. 4:**
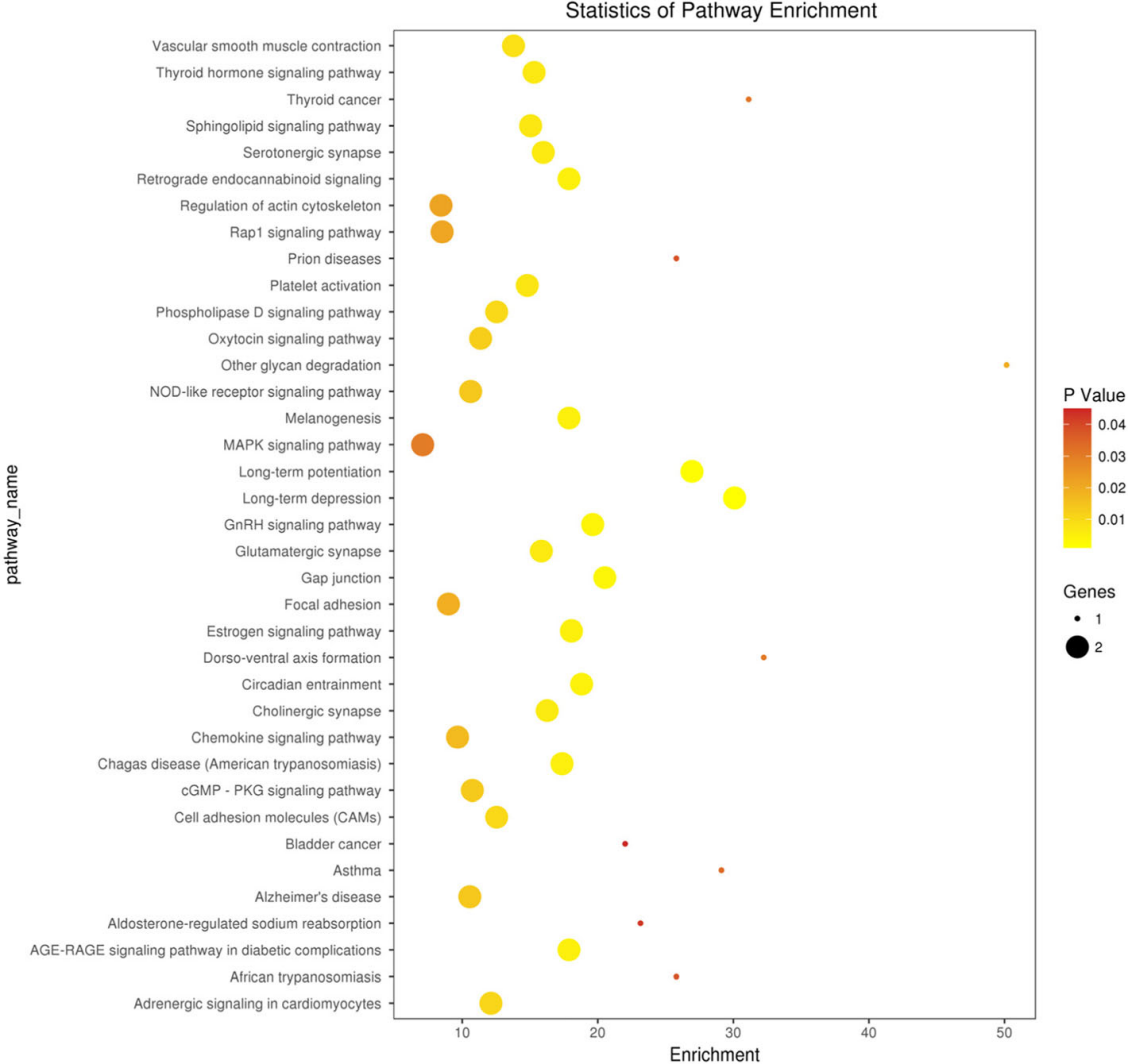
Signaling pathways of the differentially ex pressed intersection genes. miRNA prediction of intersection genes. Note:The x-axis represents the number of DEGs and the y-axis represents the pathways terms. The graph displays only significantly enriched pathways terms (*P* < 0.05) and darker yellow indicates greater significance

The GO analysis of the 30 intersection genes from the three groups revealed that the biological functions of these genes mainly included cell-cell adhesion, response to toxic substance, and lipid catabolic process (Fig. 1). The cellular components mainly included cytosol, nuclear speck, and cell-cell adhesion junction (Fig. 2). The molecular functions included cadherin binding involved in cell-cell adhesion and phospholipid binding (Fig. 3). The signaling pathways found in the KEGG database showed that these target genes are mainly distributed in the hippocampus-dependent long-term potentiation and long-term depression pathways, intercellular gap junction pathway, gonadotropin-releasing hormone signal transduction pathway, and circadian entrainment pathway (Fig. 4).

The differentially expressed mRNA was predicted using the miRanda and TargetScan software, and 2978 miRNAs were obtained by taking the intersections. After comparing groups A, B, and C, the genes with significant differences between group A and groups B and C (*PHLDB2*, *LINC01590*, *SMIM8*, *CENPT*, *VPS53*, *ITGB8*, *TTC26*, *PGM5-AS1*, *GKAP1*, *ZNF736*, and *GPATCH2*) were selected for miRNA prediction. A total of 908 miRNAs were predicted.

To analyze the miRNA-target gene regulatory network, the targeted regulatory relationship between the miRNA and target genes was used to construct the miRNA-gene interaction network. According to the screening results based on score > 175, Energe<− 35, the predicted miRNAs of the above genes included hsa-miR-4638-3p, hsa-miR-5096, hsa-miR-4763-5p, hsa-miR-1273 g-3p,hsa-miR6192, hsa-miR-4726-5p, and hsa-miR-1273a. They had higher centrality toward the genes *VPS53, PHLDB2, GPATCH2, ITGB8, ZNF736, TTC26* and *CENPT*. Among them, hsa-miR-4638-3p and hsa-miR-1273 g-3p exhibited the highest centrality (Table 2).

**Table 2:** miRNA prediction of genes with significant differences between group A and groups B and C

Gene Symbol	miRNA	Score	Energe
VPS53	hsa-miR-4638-3p	193	−45.14
	hsa-miR-5096	191	−38.75
	hsa-miR-7851-3p	184	−32.95
	hsa-miR-4763-5p	183	−38.27
	hsa-miR-2110	178	−34.93
	hsa-miR-1200	176	−29.67
	hsa-miR-619-5p	**175**	**−33.81**
ITGB8	hsa-miR-4729	179	−22.78
	hsa-miR-1208	177	−26.73
	hsa-miR-1273e	176	−29.11
	hsa-miR-136-3p	175	−21.8
PHLDB2	hsa-miR-1273 g-3p	195	−43.01
	hsa-miR6192	175	−36.3
GPATCH2	hsa-miR-1273 g-3p	195	−41.57
	hsa-miR-1273a	183	−31.32
ZNF736	hsa-miR-3189-3p	175	−31.94
TTC26	hsa-miR-1273a	187	−36.01
CENPT	hsa-miR-4726-5p	175	−35.1

Note:group A: female breast cancer patients with anthracycline-induced cardiotoxicity; group B: female breast cancer patients with chemotherapy history but no cardiotoxicity; group C: female breast cancer patients with normal ejection fraction before chemotherapy. Bold font indicates that the trends of the gene in group A were opposite to those in groups B and C. Red represents up-regulation of group A genes and down-regulation of groups B and C genes; green represents down-regulation of group A genes and up-regulation of groups B and C genes

**Fig. 5:**
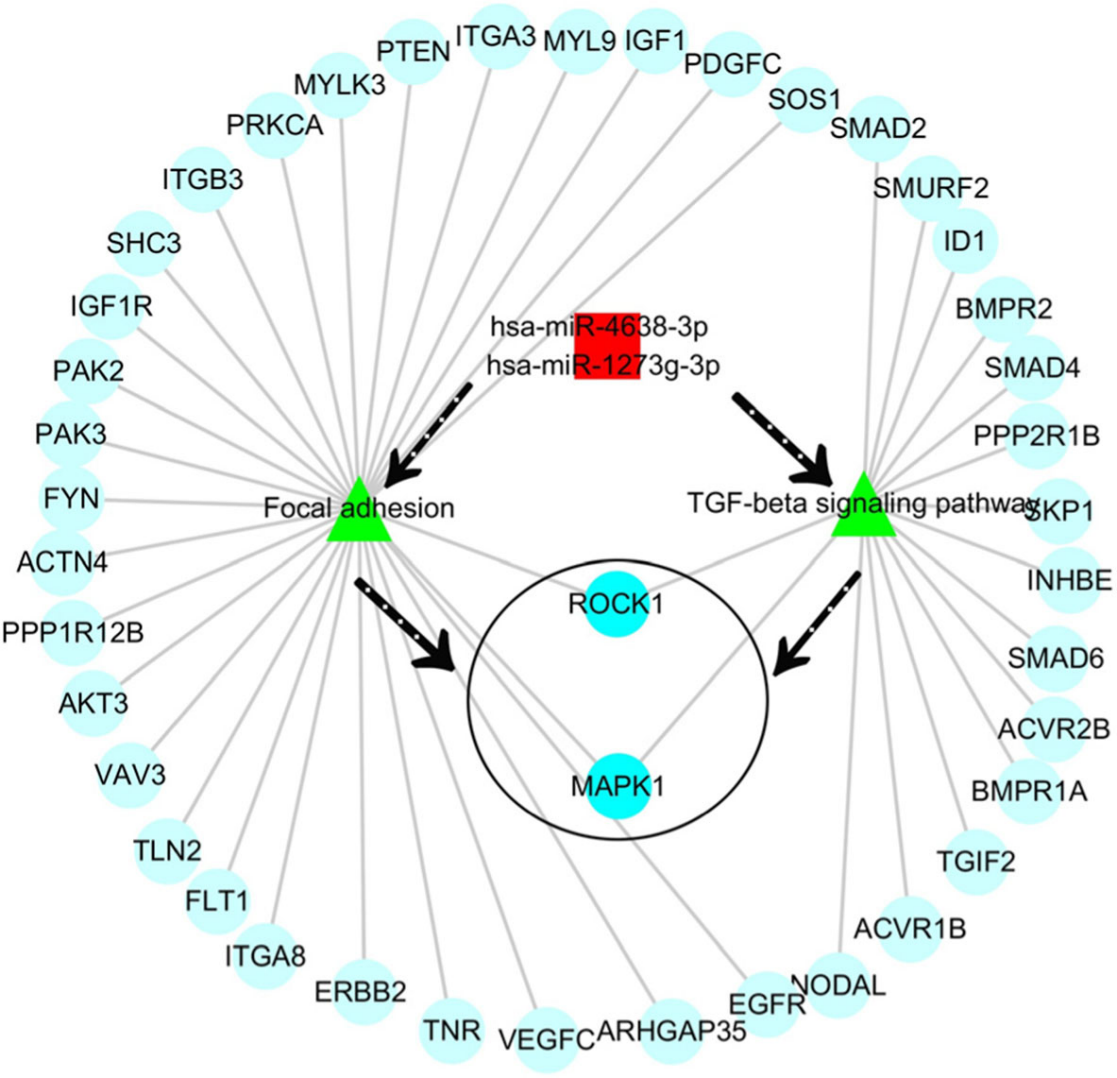
hsa-miR-4638-3p and hsa-miR-1273 g-3p Target Gene Network Map. Note:The black dotted arrow indicates the possible roles of signaling pathways and hub genes; Red represents miRNA; Green represents the signal pathway; Light blue represents potential target genes; The dark blue, that is in black circle, represents the hub gene

Entries in boldface font indicates that the trends of the gene in group A were opposite to those in groups B and C

hsa-miR-4638-3p and hsa-miR-1273 g-3p were analyzed for intersection of target genes and signaling pathways. There were 1947pcs of intersection genes of hsa-miR4638-3p and hsa-miR-1273 g-3p for signaling pathway analysis. TGF-β signaling pathway and adhesion signaling pathway were involved in cardiotoxicity, and 45pcs of intersection genes were closely related to these two pathways. ROCK1 and MAPK1 are two key genes in the pathway (Fig. 5).

### PCR verification

Demographic and baseline cardiovascular parameters are shown in Table 3.Taking U6 as the internal reference, for statistical convenience, the expression level of hsa-miR-4638-3p and hsa-miR-1273 g-3p in the peripheral blood was calculated by the 2^-△△ct^ method. Their expression levels relative to that of U6 were converted to Log_2_ ∆Ct values. The expression of hsa-miR-4638-3p and hsa-miR-1273 g-3p in the peripheral blood of the 20 subjects is presented in Table 4.

**Table 3:** Classification and Baseline Values of Patients

Group	A (after chemotherapy)	C (before chemotherapy)	*P Value*
*N*	20	20	
Age (median, range)	54 (35–75)	54 (35–75)	NS
Ejection Fractions, EF (median)	48	68	*P* < 0.001
Hypertension	0	0	NS
Diabetic	0	0	NS

Note:Group A: anthraycline based chemotherapy induced cardiotoxicity; group C:breast cancer patients ejection fraction before starting chemotherapy

**Table 4:** Expression level of hsa-miR-4638-3p and hsa-miR-1273 g-3p in the peripheral blood of each group (Log_2_^∆^Ct value)

		Mean value (*X* ± SD)	
Group	N	hsa-miR-4638-3p	hsa-miR-1273 g-3p
A	20	1.29 ± 0.72	1.00 ± 0.20
C	20	2.66 ± 1.38^*^	0.48 ± 0.17^*^
		*P =* 0.000	*P* = 0.000

Note: paired *t* test was adopted: ^*^P < 0.05. Group A: anthraycline based chemotherapy induced cardiotoxicity; group C:breast cancer patients ejection fraction before starting chemotherapy

*SD* Standard Deviation, *X* sample mean

## Discussion

The mechanism of anthracycline-induced cardiotoxicity has not been fully understood. The existing evidences reveal that it is directly related to the free radicals produced by anthracyclines [7, 8]. Unlike their mechanism of anti-tumor activity, the main mechanisms of anthracycline-induced cardiotoxicity involve iron-mediated production of reactive oxygen species (ROS) and the promotion of myocardial oxidative stress. Anthracyclines chelate iron ions and trigger the generation of oxygen free radicals, especially the hydroxyl free radicals, resulting in lipid peroxidation of myocardial cell membrane, myocardial mitochondrial DNA damage, etc. [9, 10]. The other mechanisms include the formation of toxic drug metabolites, inhibition of nucleotide and protein synthesis, release of vasoactive amines, suppression of specific gene expression, impairment of mitochondrial membrane binding, aggregation of creatine kinase activity, induction of apoptosis, interference of intracellular calcium homeostasis, changes in respiratory chain proteins, induction of nitric oxide synthase, enhancement of mitochondrial cytochrome C release, etc. [11, 12]. Other studies have shown that anthracyclines can lead to myocardial cell damage, inducing cardiac mitochondrial diseases and impairment of mitochondrial DNA and the respiratory chain in chronic cardiomyopathy [13–15]. However, a solution to this problem is yet to be identified. One of the main reasons behind this is that the mechanism of pathogenesis of anthracycline-induced diseases is not fully understood. Modern medical studies have shown that it is difficult to fully explain the origin and development of a disease using the results regarding a single gene or signaling pathway. High-throughput biological detection technology, or gene chip detection technology, can obtain a large amount of gene expression information simultaneously. We can understand the biological molecules in living cells and their interactions by analyzing such gene expression information through bioinformatic methods. This way, the key factors involved in the pathogenesis of diseases can be identified, which can serve as references for target design in clinical treatment [16].

Recent studies have shown that miRNA is involved in the processes of regulating the growth and development, mechanical remodeling, and electrical remodeling of the heart and is closely related to heart diseases [17]. The overexpression of miRNA-1 and miRNA-133 can inhibit cardiomyocyte hypertrophy [18]. Up-regulated miRNA-21 due to myocardial emergency can lead to fibroblast proliferation and myocardial interstitial fibrosis via [19]. miRNA-29, miRNA-133, and miRNA-30 have also been proved to be involved in cardiac fibrosis [20]. Terentyev et al. [21] showed that miRNA-1 can also act This enhances the release of calcium ions and promotes cardiac arrhythmogenesis. Wijnen et al. [22] evaluated the role of miRNA in myocardial fibrosis using a specific miRNA transgenic mouse model and methods such as the loss-of-function and gain-of-function.

In the present study, we used the raw data of GSE40447 gene chip in the GEO database and adopted software packages to analyze differentially expressed miRNAs. The DGE analysis between group A and groups B and C showed that there were 30 intersection genes. With further analysis of the biological functions and signaling pathways, it was found that the main functions of the differentially expressed miRNA-mRNA included cell-cell adhesion, response to toxic substance, and lipid catabolic process. Their cellular components mainly included cytosol, nuclear speck, and cell-cell adhesion junction. The genes *VPS53, ITGB8, ZNF736,* and *TTC26* play important roles in cholesterol transport in cardiac cells, mediating the interaction between cell-cell and cell-extracellular matrix, regulate transcription, and transport proteins [23–25]. Among them *VPS53* and *PHLDB2, GPATCH2, ITGB8, ZNF736, TTC26* exhibited opposite expressions in groups A, B, and C. This suggests that *VPS53* and *PHLDB2, GPATCH2, ITGB8, ZNF736, TTC26* might have opposite effects on some processes of the disease. The molecular functions included cadherin binding involved in cell-cell adhesion and phospholipid binding. The signaling pathways found in the KEGG database showed that these target genes were mainly distributed in the hippocampus-dependent long-term potentiation and long-term depression pathways, intercellular gap junction pathway, gonadotropin-releasing hormone signal transduction pathway, and circadian entrainment pathway. The biological functions of miRNA were mainly realized through the regulation and control of their target genes. The analysis of the functions of these differentially expressed miRNAs revealed that hsa-miR-4638-3p, hsa-miR-5096, hsa-miR-4763-5p, hsa-miR-1273 g-3p, hsa-miR6192, hsa-miR-4726-5p, and hsa-miR-1273a have higher centrality. This indicates that these miRNA targets are extensive, including oncogenes, tumor suppressor genes, signal transduction genes, and cell cycle regulation genes. Therefore, target gene prediction can provide a theoretical basis for further target gene verification experiments and avoid blind obedience. Further analysis of the miRNA-target gene regulatory network also showed that the miRNAs such as hsa-miR-4638-3p and hsa-miR-1273 g-3p had the highest centrality to the surrounding target genes, suggesting that they are the core regulatory miRNAs. The PCR results were consistent with the predicted results. The results of clinical sample validation showed that there was a significant difference between hsa-miR4638-3p and hsa-1273 g-3p before and after anthracycline chemotherapy. According to GO annotation and signal pathway analysis, Rho Associated Coiled-coil Containing Protein Kinase 1(ROCK1) and Mitogen-Activated Protein Kinase 1(MAPK1) are two key genes in the pathway, which may regulate the cardiac toxicity of anthracycline drugs through TGF-β signaling pathway and adhesion signaling pathway. Rho-Associated Coiled-Protein Kinase (ROCK) has serine/threonine protein kinase activity. It is a Rho-binding protein associated with apoptosis, which is also the main molecule of the Rho family. The Ras Homolog C/Rho-Associated Coiled-Protein Kinase (RHO/ROCK) pathway plays an important role in mediating various cellular functions, including contraction, actin cytoskeleton, cell adhesion and movement, proliferation, cytokinesis, and gene expression, all of which are involved in the pathogenesis of cardiovascular disease [26, 27]. RHO/ROCK specific inhibitors show promise in the prevention of heart disease. ROCK1 is activated after cardiomyocytes are treated with doxorubicin in vitro, and RHO/ROCK inhibitors can prevent doxorubicin cardiotoxicity [28]. ROCK1 mediates autophagy dysregulation and apoptosis in adriamycin cardiotoxicity, and promotes cardiac remodeling and reverse dysfunction [29]. MAPK1 activation provides a possible mechanism to prevent doxorubicin-induced cardiomyocyte injury [30–32]. These findings suggested that whether specific miRNAs inhibitors or activators, specific pathway inhibitors or activators can be used to alleviate cardiotoxicity caused by anthracyclines; whether certain natural compounds can achieve heart protection by regulating specific miRNAs and target gene expression; Whether the detection of heart-specific miRNA in plasma can be used to identify patients with subclinical cardiotoxicity and prevent severe complications of anthracycline drugs. Therefore, miRNAs have considerable research value in cardiotoxicity caused by anthracyclines.

The present study had some limitations. Based on the results of bioinformatic analysis, we can predict numerous target genes. However, there are also some false positives; therefore, its accuracy needs further validation by molecular biology experiment, in order to identify the real target genes of each miRNA and to verify the exact role played by miRNA in the development and incidence of anthracycline-induced cardiac toxicity. In the verification experiment, The number of cases collected in this experiment is small, and the experimental results have certain limitations. The reliability of the data needs to be further expanded to confirm the sample size.

## Conclusion

Collectively, the current study identified two pivotal miRNAs, hsa-miR-4638-3p and hsa-miR-1273 g-3p, They are closely related to the cardiotoxicity induced by anthracyclines. Functional enrichment analysis indicated that mainly included cell-cell adhesion, response to toxic substance, and lipid catabolic process. However, the results of the present study are based entirely on bioinformatics analyses and lack in vivo and in vitro experimental evidence. Further research is required to delineate their potential roles in Anthracycline Cardiotoxicity.

## Data Availability

The data used to support the findings of this study are available from the corresponding author upon request.

## Abbreviations

CaMK II: Ca^2+^/calmodulin-dependent protein kinase II
cDNA: Complementary DNA
*CENPT*: Centromere protein T
EF: Ejection fraction
FC: Fold change
FDR: False discovery rate
*GKAP1*: G kinase anchoring protein 1
GO: Gene ontology
*GPATCH2*: G-patch domain containing 2
IQR: Interquartile range
*ITGB8*: Integrin subunit beta 8
KEGG: Kyoto encyclopedia of genes and genomes
*LINC01590*: Long intergenic non-protein coding RNA 1590
MAPK1: Mitogen-activated protein kinase 1
miRNA: MicroRNA
*PGM5-AS1*: PGM5 antisense RNA 1
*PHLDB2*: Pleckstrin homology like domain family B member 2
*PS53*: VPS53 subunit of GARP complex
q-PCR: Quantitative-polymerase chain reaction
*ROCK1*: Rho associated coiled-coil containing protein kinase 1
*SMIM8*: Small integral membrane protein 8
*SPRY1*: Sprouty RTK signaling antagonist 1
TGFBR: Transforming growth factor beta
*TTC26*: Tetratricopeptide repeat domain 26
*ZNF736*: Zinc finger protein 736
